# The Efficacy and Central Remodeling Mechanism of a Composite TMS Pattern in First-Episode and Recurrent Depressive Disorders

**DOI:** 10.3390/brainsci15080801

**Published:** 2025-07-28

**Authors:** Li Pu, Jiang Wu, Shan Huang, Dandan Liu, Xi Tan, Hongmei Yan, Guojian Yan, Dezhong Yao

**Affiliations:** The Clinical Hospital of Chengdu Brain Science Institute, MOE Key Laboratory for Neuroinformation, School of Life Science and Technology, University of Electronic Science and Technology of China, Chengdu 610036, China; 202311140635@std.uestc.edu.cn (L.P.); wj_uest@163.com (J.W.); 18708207423@163.com (S.H.); 19102899430@163.com (D.L.); tan_xi1234@163.com (X.T.); guojianyan_uestc@163.com (G.Y.); dyao@uestc.edu.cn (D.Y.)

**Keywords:** depressive disorders, transcranial magnetic stimulation, intermittent theta burst stimulation, low-frequency stimulation, event-related potentials

## Abstract

**Background:** This study aims to evaluate the efficacy of a combined transcranial magnetic stimulation (TMS) protocol incorporating intermittent theta burst stimulation (iTBS) and low-frequency TMS in adults diagnosed with first-episode and recurrent depressive disorders. **Methods:** A prospective, double-blind, parallel-group trial was conducted involving 42 participants (21 with first-episode depressive disorder and 21 with recurrent depressive disorder) recruited from Chengdu, China. All subjects received 10 sessions of TMS over two weeks. The primary outcome measure was suicidal ideation, assessed using the Beck scale for suicide ideation. Secondary outcomes included sleep quality, depressive symptoms, anhedonia, and cognitive function. Event-related potentials (ERPs) were also recorded. Data were analyzed using SPSS V.21.0, with statistical significance defined as *p* < 0.05. **Results:** Both patient groups exhibited significant reductions in suicidal ideation following the composite TMS intervention. Secondary outcomes showed significant improvements in sleep quality, overall depressive symptoms, anhedonia, and cognitive function. Notably, a significant association was found between improvements in sleep quality and depressive symptoms in the first-episode group, suggesting differential underlying mechanisms compared to recurrent depression. **Limitations:** The relatively short intervention and follow-up period limits the ability to assess the long-term sustainability of the observed benefits. Future studies with extended follow-up periods are warranted to evaluate the persistence of TMS effects and the potential need for maintenance sessions. **Conclusions:** The combined protocol of iTBS and low-frequency TMS effectively reduces suicidal ideation and improves various clinical outcomes in both first-episode and recurrent depressive disorders, indicating the effectiveness of the physical intervention, especially for the first-episode patients. These findings underscore the importance of personalized treatment strategies based on the clinical history of depressive episodes. Further research with longer follow-up periods is warranted to assess the long-term sustainability of TMS effects.

## 1. Introduction

Depressive disorders, characterized by persistent feelings of sadness and a lack of interest or pleasure in previously rewarding or enjoyable activities, represent a significant public health concern globally. They are associated with substantial morbidity, mortality, and economic burden due to their chronic nature and recurrence rates [1]. Among various therapeutic approaches, transcranial magnetic stimulation (TMS) has emerged as a promising non-invasive method for modulating neuronal activity and alleviating symptoms in patients with major depressive disorder (MDD) [2].

In recent years, advancements in TMS protocols have facilitated the development of more targeted and effective treatments for depression. Intermittent theta burst stimulation (iTBS) has been shown to induce rapid and long-lasting antidepressant effects by modulating cortical excitability and synaptic plasticity [3]. Additionally, low-frequency TMS on the right dorsolateral prefrontal cortex (DLPFC) has been found to suppress hyperactivity in this region, which is often associated with depressive symptoms [4]. Combining these two approaches—targeting the left DLPFC with iTBS and the right DLPFC with low-frequency stimulation—may produce synergistic effects by simultaneously modulating multiple neural pathways involved in depression [5].

Despite the promising results of these individual TMS protocols, there is limited research directly comparing their combined efficacy in patients with first-episode versus recurrent depressive disorders. Understanding these differences is crucial as it can inform personalized treatment strategies and improve clinical outcomes. For example, first-episode patients might exhibit distinct responses to TMS interventions when compared with those with recurrent episodes due to varying underlying pathophysiological mechanisms and neuroplastic changes.

This study aims to fill this gap by conducting a prospective, double-blind, parallel-group trial to evaluate the efficacy of the combined iTBS and low-frequency TMS protocol in adults diagnosed with first-episode and recurrent depressive disorders. Our primary objective was to assess changes in suicidal ideation, while secondary objectives included evaluating improvements in sleep quality, overall depressive symptoms, anhedonia, and cognitive function. By examining the relationship between improvements in sleep quality and depressive symptoms, particularly in the first-episode group, we aim to better understand the underlying mechanisms and potential predictors of treatment response.

We hypothesized that both patient groups would exhibit significant improvements in the primary and secondary outcome measures, as well as event-related potentials, following the 2-week combined TMS intervention. Importantly, we further hypothesized that the magnitude of these improvements would differ between the two groups, suggesting differential responsiveness to the same neuromodulation protocol in the first-episode versus recurrent depressive disorders, potentially reflecting differential underlying pathophysiological mechanisms and potential implications for personalized treatment strategies [6].

The study’s rigorous design, including blinding of treating therapists and outcome assessors, and the use of standardized and validated assessment tools, ensures the reliability and validity of the findings. By providing new insights into the efficacy of TMS protocols in different depressive disorder subtypes, this research contributes to the optimization of treatment strategies and the enhancement of clinical outcomes for patients with depressive disorder.

## 2. Materials and Methods

### 2.1. Study Subjects

The 42 subjects in this study (21 patients with first-episode depressive disorder and 21 patients with recurrent depressive disorder) were recruited from February 2024 to June 2024 in Chengdu, China. The study was approved by the ethics committee of Chengdu 4th People’s Hospital, and the clinical trial registration ID is NCT06417437. All participants provided written informed consent before any procedures were performed.

The inclusion criteria for the depressive disorder participants were as follows: (1) Meet the DSM-V diagnostic criteria for “Depressive Disorders” [7]; (2) Han ethnicity, age ≥ 18 years; (3) Junior high school education level or above; (4) right-handed; (5) HAMD-17 score ≥ 17; (6) medication use: No use of psychiatric-related medications in the past 3 months or a stable medication regimen for the past 3 months and with antidepressant medications limited to selective serotonin reuptake inhibitors (SSRIs) and serotonin and norepinephrine reuptake inhibitors (SNRIs) during physical regulation; and (7) willing to participate in the experiment, accept treatment, and sign an informed consent form. According to whether it is a first episode, depressive disorder participants meeting the inclusion criteria will be divided into a first episode group and a recurrent group.

The exclusion criteria for all individuals were as follows: (1) Combined or previous history of organic brain diseases or severe brain trauma, personal or family history of epilepsy; (2) severe cardiac, hepatic, or renal dysfunction; (3) patients with severe physical diseases; (4) history of substance dependence or abuse (alcohol, cocaine, drugs, etc.); (5) concurrent other psychiatric disorders; (6) pregnancy or lactation; (7) use of electroconvulsive therapy (ECT) without seizure, transcranial magnetic stimulation (TMS), or other physical treatments within the past six months; (8) implanted autonomic nerve stimulators; (9) implanted electronic or metal devices (such as pacemakers, defibrillators, stents, orthopedic steel plates, etc.), or those who have undergone ventriculoperitoneal shunting; and (10) significant visual or auditory impairments that prevent completion of neuropsychological testing and scale assessments.

### 2.2. Clinical and Psychological Evaluation

Demographic data were collected at baseline, and clinical and psychological assessments were conducted both at baseline and at the end of the 2-week treatment. Two senior psychology experts, each with >10 years of work experience, independently performed the psychological evaluation without knowledge of the participants’ clinical diagnosis, then another senior psychiatrist reviewed the assessment results. The following scales were used, including the following: the Pittsburgh sleep quality index [8], the childhood trauma questionnaire [2], the trail making test [9], the digit span test [5], the Hamilton anxiety rating scale [10], the Hamilton depression rating scale [1], the social support rating scale [11], the Beck scale for suicide ideation [6], the Snaith–Hamilton pleasure scale [12], the emotion regulation score [12], a self-rating anxiety scale [13] and a self-rating depression scale [14]. The Pittsburgh sleep quality index was used to evaluate sleep quality. It consists of 19 items, yielding a global score ranging from 0 to 21, with higher scores indicating poorer sleep quality. The childhood trauma questionnaire was used to assess experiences of child abuse and neglect. Each item was rated on a 5-point Likert scale ranging from 1 (never true) to 5 (very often true). The trail making test is a measure of attention and executive functions. The digit span test is a measure of working memory capacity. The Hamilton anxiety rating scale and Hamilton depression rating scale were used to assess the severity of anxiety and depressive symptoms in participants. The social support rating scale is a widely used instrument for assessing the level of social support. The Beck scale for suicide ideation was used to assess the level of suicidal ideation. All participants were required to complete the first five screening items. If both Item 4 and Item 5 were endorsed as “No,” the participant was considered to have no suicidal ideation and the assessment was terminated at that point. However, if either Item 4 or Item 5 was rated as “Weak” or “Moderate to Strong,” the presence of suicidal ideation was established, and the participant proceeded to complete the remaining 14 items. The level of suicide risk was determined based on the total score from Items 6 to 19. The Snaith–Hamilton pleasure scale is used to assess anhedonia. The emotion regulation score is a self-report questionnaire to evaluate the emotion regulation capacity. The self-rating anxiety scale and self-rating depression scale were used to assess anxiety and depression, respectively.

### 2.3. Event-Related Potentials (ERPs)

ERPs were recorded using MEB-23120 electromyogram/evoked potential equipment (Neuropack, Nihon Koden, Kyoto, Japan). The recording electrode was positioned at Cz according to the 10/20 system, and the reference electrodes were placed at the bilateral mastoids.

MMN was elicited using an auditory oddball paradigm through headphones. Standard stimuli (60 dB nHL, 1000 Hz tones, with an envelope of 10 ms rise, 100 ms plateau, and 10 ms fall) and deviant stimuli (80 dB nHL, 2000 Hz tones, same envelope) were presented in a randomized sequence at an 80%:20% ratio and 1 Hz frequency. Signals were averaged over 40 trials. MMN was obtained by subtracting the responses to standard stimuli from those to deviant stimuli, with subjects remaining relaxed with eyes closed.

P300 was recorded under the same oddball paradigm, with signals averaged over 30 trials and digitally filtered with a bandpass of 0.1 to 100 Hz. Subjects were instructed to press a button in response to target stimuli. The analysis time and sensitivity were 100 ms/div and 20 μV/div, respectively. ERP measurements were taken at baseline and after two weeks for all subjects.

### 2.4. Blinding

Treating therapists were blinded to treatment allocation, and outcome assessors were blinded to treatment assignment. Prior to the start of the study, they were explicitly instructed not to discuss participants’ diagnostic information either with the participants themselves or with any individuals who were aware of the group assignments.

### 2.5. Interventions

In this study, we utilized the YRD CCY-1 transcranial magnetic stimulation device manufactured by Wuhan Yiruide Company, Wuhan, China. All patients received 10 trials (5 trials a week for 2 weeks). Each patient’s resting motor threshold (RMT) was tested in accordance with the standard practice. Both groups employed a combined protocol of iTBS on the left dorsolateral prefrontal cortex (DLPFC) and low-frequency stimulation on the right DLPFC, based on a previously published study [15]. The specific protocols are as follows:

The left DLPFC was stimulated using intermittent theta burst stimulation (iTBS) with an ‘8’-shaped coil placed parallel to the skull and in close contact with the scalp, at 90% of RMT. Stimulation was administered once daily, totaling 600 pulses per session, with a duration of 3 min and 20 s.

For the right DLPFC, 1 Hz low-frequency stimulation was administered using the same ‘8’-shaped coil configuration placed parallel to the skull and in close contact with the scalp, at 100% of RMT. Stimulation was administered once daily, totaling 1200 pulses per session, with a duration of 21 min.

### 2.6. Outcome Measures

The primary outcome measure was the Beck scale for suicide ideation in the past week (1a + 2a……19a) after 2 weeks of treatment. Secondary outcome measures included the Pittsburgh sleep quality index, childhood trauma questionnaire, trail making test, digit span test, Hamilton anxiety rating scale, Hamilton depression rating scale, social support rating scale, Snaith–Hamilton pleasure scale, emotion regulation score, self-rating anxiety scale and self-rating depression scale.

### 2.7. Safety Measures

Adverse effects and accidents were closely monitored and recorded throughout the intervention period. Serious adverse events (SAEs) and instances of treatment discontinuation were documented and assessed for their relevance to the clinical interventions.

### 2.8. Statistical Analysis

Statistical analyses for the clinical data were conducted using SPSS software, version 21.0 (SPSS, Chicago, IL, USA). Continuous variables were expressed as mean ± standard deviation (SD). An independent sample *t*-test was used to compare two groups when the data conformed to normal distribution and homogeneity of variance.

For comparisons between two groups before and after treatment, repeated measures analysis of variance (ANOVA) was performed. The Greenhouse–Geisser correction was applied for the test of within-subject effects, and post hoc tests were conducted using the least significant difference (LSD) method. When the data did not conform to normal distribution or homogeneity of variance, non-parametric tests were used. Statistical significance was set at *p* < 0.05 (two-sided). Partial correlation analysis, controlling for age, was performed to investigate the associations between changes in the Pittsburgh sleep quality index and the Hamilton depression rating scale in the first-episode group, in order to minimize potential confounding effects associated with recurrent depressive disorder, such as medication history and cumulative depressive episodes. By focusing on the first-episode group, we aimed to capture the more direct associations between these indicators under relatively homogeneous clinical conditions. A significance level of *p* < 0.05 was used for the partial correlation analysis.

## 3. Results

### 3.1. Characteristics of Participants

The clinical characteristics of the participants are summarized in Table 1. The patients with first-episode depressive disorder and patients with recurrent depressive disorder differed significantly in age (*p* < 0.05), but not in other baseline characteristics, cognitive performance, or emotional state (*p* > 0.05) (Table 1).

### 3.2. Efficacy Outcomes

#### 3.2.1. Primary Outcome

**Figure 1:**
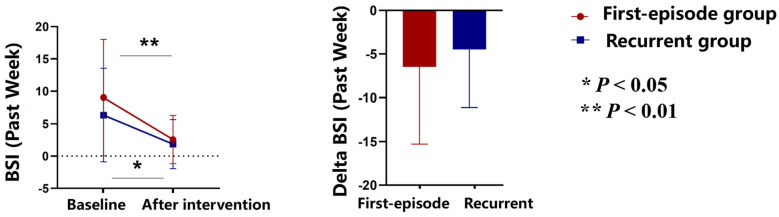
Primary outcome. BSI (Past Week) scores before and after 2-week TMS intervention in first-episode and recurrent depressive disorder groups. Both groups showed significant reductions in BSI scores (*p* < 0.05, *p* < 0.01).

At baseline, the mean Beck scale for suicide ideation in the past week was 9.05 (8.98) in the first-episode depressive disorder group and 6.33 (7.21) in the recurrent depressive disorder group. After 2-week TMS intervention, the mean Beck scale for suicide ideation in the past week was 2.57 (3.74) (*p* = 0.001) in the first-episode depressive disorder group and 1.86 (3.80) (*p* = 0.025) in the recurrent depressive disorder group (Figure 1).

#### 3.2.2. Secondary Outcomes

**Figure 2:**
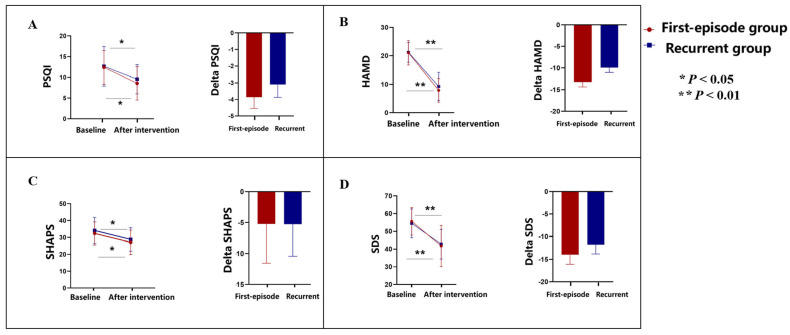
(**A**–**D**) Secondary outcomes. Changes in clinical measures before and after 2-week TMS intervention in first-episode and recurrent depressive disorder groups. (**A**) Pittsburgh Sleep Quality Index (PSQI); (**B**) Hamilton Depression Rating Scale (HAMD); (**C**) Snaith–Hamilton Pleasure Scale (SHAPS); (**D**) Self-rating Depression Scale (SDS). All measures showed significant improvement after intervention in both groups (*p* < 0.05, *p* < 0.01).

At baseline, the mean Pittsburgh sleep quality index was 12.43 (4.06) in the first-episode depressive disorder group and 12.67 (4.77) in the recurrent depressive disorder group. After 2-week TMS intervention, the mean Pittsburgh sleep quality index was 8.57 (4.03) (*p* = 0.003) in the first-episode depressive disorder group and 9.57 (3.53) (*p* = 0.017) in the recurrent depressive disorder group (Figure 2A).

At baseline, the mean Hamilton depression rating scale was 21.10 (4.32) in the first-episode depressive disorder group and 21.24 (3.52) in the recurrent depressive disorder group. After 2-week TMS intervention, the mean Hamilton depression rating scale was 7.90 (4.21) (*p* = 0.007) in the first-episode depressive disorder group and 9.29 (4.95) (*p* = 0.008) in the recurrent depressive disorder group (Figure 2B).

At baseline, the mean Snaith–Hamilton pleasure scale was 32.33 (6.80) in the first-episode depressive disorder group and 34.10 (7.77) in the recurrent depressive disorder group. After 2-week TMS intervention, the mean Snaith–Hamilton pleasure scale was 27.10 (7.22) (*p* = 0.021) in the first-episode depressive disorder group and 28.81 (6.91) (*p* = 0.019) in the recurrent depressive disorder group (Figure 2C).

At baseline, the mean self-rating depression scale was 55.67 (7.80) in the first-episode depressive disorder group and 54.52 (8.18) in the recurrent depressive disorder group. After 2-week TMS intervention, the mean self-rating depression scale was 41.71 (11.61) (*p* = 0.005) in the first-episode depressive disorder group and 42.76 (8.35) (*p* = 0.009) in the recurrent depressive disorder group (Figure 2D).

### 3.3. Correlation Between Changes of Pittsburgh Sleep Quality Index and Hamilton Depression Rating Scale in the First-Episode Group

We observed a decrease in the Pittsburgh sleep quality index [−3.86 (3.10)] in the first-episode depressive disorder group from baseline to the conclusion of the 2-week intervention period. We also observed a decrease in the Hamilton depression rating scale [−13.19 (5.19) vs. −11.95 (3.87)] in the first-episode depressive disorder group from baseline to the conclusion of the 2-week intervention period. After adjusting for age, the partial correlation analysis showed a significant association between the changes of Pittsburgh sleep quality index and Hamilton depression rating scale in the first-episode depressive disorder group (R = 0.572, *p* = 0.008).

### 3.4. Event-Related Potentials

**Figure 3:**
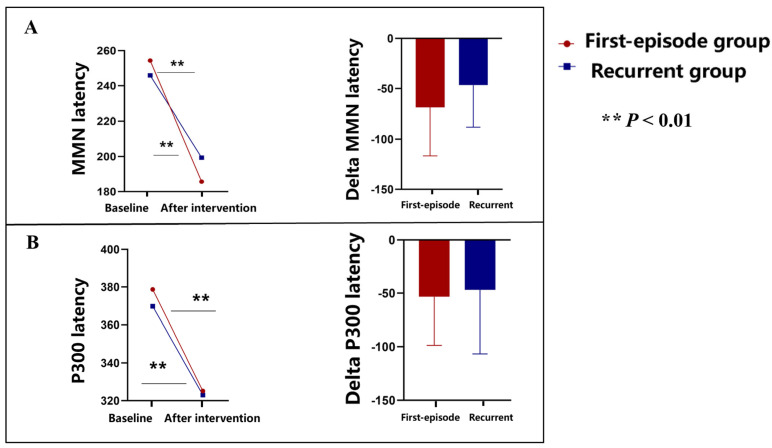
(**A**,**B**) Event-related potentials. Changes in MMN and P300 latencies before and after 2-week TMS intervention in first-episode and recurrent depressive disorder groups. (**A**) Mismatch negativity (MMN) latency; (**B**) P300 latency. Both MMN and P300 latencies significantly decreased after intervention in both groups (*p* < 0.01).

At baseline, the mean MMN latency was 254.38 (47.81) in the first-episode depressive disorder group and 245.90 (36.64) in the recurrent depressive disorder group. After 2-week TMS intervention, the mean MMN latency was 185.71 (22.94) (*p* = 0.007) in the first-episode depressive disorder group and 199.38 (33.85) (*p* = 0.009) in the recurrent depressive disorder group (Figure 3A).

At baseline, the mean P300 latency was 378.67 (44.30) in the first-episode depressive disorder group and 369.76 (52.05) in the recurrent depressive disorder group. After 2-week TMS intervention, the mean P300 latency was 325.19 (24.86) (*p* = 0.003) in the first-episode depressive disorder group and 323.00 (32.786) (*p* = 0.005) in the recurrent depressive disorder group (Figure 3B).

## 4. Discussion

The findings from our study provide compelling evidence supporting the efficacy of combined iTBS and low-frequency TMS protocols in reducing suicidal ideation and improving various clinical outcomes in patients with depressive disorders. Both first-episode and recurrent depressive disorder groups showed significant reductions in Beck scale scores for suicidal ideation after the 2-week intervention, highlighting the potential of TMS as a critical component in the therapeutic arsenal for MDD [10].

Our results also demonstrate significant improvements in secondary outcomes, including sleep quality, overall depressive symptoms, anhedonia, and cognitive function, across both patient groups. These findings align with previous research that has reported the benefits of TMS in enhancing mood and cognitive performance by modulating neural circuits implicated in depression. For example, one study [11] demonstrated that TMS could effectively improve cognitive function and reduce depressive symptoms, which is consistent with our findings of decreased scores on the Hamilton depression rating scale and the Snaith–Hamilton pleasure scale.

One of the most intriguing aspects of our study was the observed correlation between improvements in sleep quality and depressive symptoms in the first-episode depressive disorder group. This finding suggests that early intervention in depressive episodes may yield more pronounced benefits in sleep regulation and overall symptomatology, potentially due to less entrenched pathophysiological changes compared with recurrent episodes [8]. The significant association between Pittsburgh sleep quality index changes and Hamilton depression rating scale reductions in the first-episode group underscores the importance of addressing sleep disturbances as a critical component of depressive disorder treatment.

It is important to note that the two patient groups did not exhibit significant between-group differences in the primary and secondary outcome measures, as well as in ERPs, following the combined TMS intervention. This may suggest that the combined TMS protocol produced comparable effects in patients with first-episode and recurrent depressive disorders, despite their differing clinical histories. These findings imply that such a neuromodulation strategy may exert broad therapeutic effects across subtypes of depressive disorders.

The significant reductions in MMN and P300 latencies observed in both the First-episode depressive disorder group and the recurrent depressive disorder group after a 2-week TMS intervention suggest notable improvements in neural processing speed and cognitive function. These findings align with previous research indicating that TMS can enhance neural plasticity and functional connectivity in the brain, particularly in regions implicated in cognitive and emotional regulation [16]. The decrease in MMN latency indicates enhanced pre-attentive auditory processing and sensory memory, reflecting a normalization of disrupted auditory processing pathways, which are frequently impaired in depressive disorders [17]. Similarly, the reduction in P300 latency suggests improved cognitive function, particularly in attention and working memory processes [18]. As the P300 component is associated with attentional resource allocation and the updating of working memory, its latency reduction indicates a more efficient cognitive processing mechanism and improved neural efficiency [19].

Mechanistically, TMS is believed to modulate synaptic plasticity through long-term potentiation (LTP) and long-term depression (LTD) of synapses, which are critical for memory and learning processes [20]. This modulation likely underpins the observed improvements in MMN and P300 latencies. By repeatedly stimulating specific brain regions, TMS may enhance the excitability of cortical neurons, thereby strengthening neural networks involved in sensory processing and cognitive functions [21]. The differences observed between the first-episode and recurrent groups, with the first-episode group showing more pronounced reductions in latencies, may be due to greater neural plasticity in patients experiencing their first episode compared with those with recurrent episodes, whose neural pathways might be more resistant to change due to chronicity and cumulative effects of the disorder. These findings underscore the potential of TMS as a therapeutic modality to enhance cognitive processing and neural efficiency in depressive disorders, providing a basis for personalized treatment strategies that target specific neural mechanisms underlying cognitive dysfunction in depression [22,23,24].

Despite the promising results, several limitations of our study should be acknowledged. Firstly, the study did not include a true control group, which limits the ability to attribute the observed effects solely to the intervention. Non-specific factors cannot be ruled out. Future randomized controlled trials are warranted to validate the efficacy of the intervention. Additionally, repeated assessments during the treatment course are needed to better characterize the temporal dynamics of treatment response. Secondly, the relatively short duration of the intervention and follow-up period limits the ability to assess the long-term sustainability of the observed benefits. Future studies with extended follow-up periods are warranted to evaluate the persistence of TMS effects and the potential need for maintenance sessions. Thirdly, while the study was adequately powered to detect significant changes in primary and secondary outcomes, larger sample sizes would enhance the generalizability of the findings and allow for more detailed subgroup analyses. Moreover, although this study involved EEG, it only analyzed relatively simple waveform indicators such as MMN and P300. In the future, more electrode channels can be used to analyze more characteristic waveforms, revealing the mechanisms of intervention and the differences between first-episode and recurrent cases from various perspectives such as brain networks and functional connectivity. This would provide evidence for personalized diagnosis and treatment. Lastly, participants’ diagnoses might have been unintentionally disclosed during the study, possibly compromising the blinding of TMS operators and outcome assessors. This constitutes a potential limitation.

## 5. Conclusions

While limited by the absence of a control group, the findings of this study provide preliminary evidence that a combined iTBS and low-frequency TMS protocols may help reduce suicidal ideation and improve a range of clinical outcomes in patients with first-episode and recurrent depressive disorders efficiency. The differential response patterns observed highlight the need for personalized treatment strategies that consider the clinical characteristics, especially sleep disturbances. By advancing our understanding of the mechanisms and efficacy of TMS in diverse depressive disorder subtypes, this research contributes to the development of optimized and more effective therapeutic approaches for MDD.

## Figures and Tables

**Table 1:** Comparison of baseline characteristics between first-episode and recurrent MDD groups at baseline.

	MDD Group (n = 42)	MDD Group		*p* Value
		First-Episode Group (n = 21)	Recurrent Group (n = 21)	
Gender (Female, %)	28 (66.67)	15 (71.43)	13 (61.91)	0.74
Age (years)	31.07 (9.20)	26.1 (6.07)	36.05 (9.21)	**<0.001**
Height (cm)	163.71 (8.47)	164.33 (8.14)	163.10 (8.95)	0.64
Weight (kg)	59.27 (12.93)	57.95 (11.49)	60.60 (14.39)	0.52
Smoking (%)	12 (28.57)	6 (28.57)	6 (28.57)	0.48
Alcohol consumption (%)	19 (45.24)	11 (52.39)	8 (38.10)	0.54
Family history of psychiatric disorders (%)	1 (2.38)	1 (4.76)	0 (0)	1.00
Pittsburgh sleep quality index	12.55 (4.37)	12.43 (4.06)	12.67 (4.77)	0.86
Childhood trauma questionnaire	54.48 (6.77)	55.57 (5.29)	53.38 (7.97)	0.30
Trail making test-A	31.58 (11.96)	31.39 (11.94)	31.78 (11.94)	0.92
Trail making test-B	86.84 (32.16)	80.68 (31.85)	93.01 (32.04)	0.22
Digit span test-forward	7.64 (1.38)	8.05 (1.43)	7.24 (1.22)	0.06
Digit span test—backward	4.55 (1.71)	4.86 (1.71)	4.24 (1.70)	0.25
Hamilton anxiety rating scale	15.81 (5.61)	14.71 (5.51)	16.90 (5.64)	0.21
Hamilton depression rating scale	21.17 (3.89)	21.10 (4.32)	21.24 (3.52)	0.91
Social support rating scale—objective support	7.14 (2.86)	7.19 (2.50)	7.10 (3.24)	0.92
Social support rating scale—subjective support	16.10 (4.84)	16.14 (4.40)	16.05 (5.35)	0.95
Social support rating scale—utilization of Support	6.43 (1.47)	6.81 (1.57)	6.05 (1.28)	0.09
Beck Scale for suicide ideation in the past week	7.69 (8.16)	9.05 (8.98)	6.33 (7.21)	0.17
Snaith–Hamilton pleasure scale	33.21 (7.26)	32.33 (6.80)	34.10 (7.77)	0.44
Emotion regulation score	42.45 (9.25)	42.71 (8.71)	42.19 (9.96)	0.86
Self-rating anxiety scale	46.64 (9.97)	48.05 (9.04)	45.24 (10.86)	0.37
Self-rating depression scale	55.10 (7.92)	55.67 (7.80)	54.52 (8.18)	0.65

Note: *p*-value represents the statistical significance of differences in baseline characteristics between the first-episode group and the recurrent episode group.

## Data Availability

The raw data supporting the conclusions of this article will be made available by the authors on request.
